# Membrane tension-mediated stiff and soft tumor subtypes closely associated with prognosis for prostate cancer patients

**DOI:** 10.1186/s40001-023-01132-4

**Published:** 2023-05-13

**Authors:** Dechao Feng, Jie Wang, Xu Shi, Dengxiong Li, Wuran Wei, Ping Han

**Affiliations:** Department of Urology, Institute of Urology, West China Hospital, Sichuan University, Guoxue Xiang #37, Chengdu, 610041 Sichuan People’s Republic of China

**Keywords:** Prostate cancer, Biochemical recurrence, Membrane tension, Nonnegative matrix factorization

## Abstract

**Background:**

Prostate cancer (PCa) is usually considered as cold tumor. Malignancy is associated with cell mechanic changes that contribute to extensive cell deformation required for metastatic dissemination. Thus, we established stiff and soft tumor subtypes for PCa patients from perspective of membrane tension.

**Methods:**

Nonnegative matrix factorization algorithm was used to identify molecular subtypes. We completed analyses using software R 3.6.3 and its suitable packages.

**Results:**

We constructed stiff and soft tumor subtypes using eight membrane tension-related genes through lasso regression and nonnegative matrix factorization analyses. We found that patients in stiff subtype were more prone to biochemical recurrence than those in soft subtype (HR 16.18; *p* < 0.001), which was externally validated in other three cohorts. The top ten mutation genes between stiff and soft subtypes were DNAH, NYNRIN, PTCHD4, WNK1, ARFGEF1, HRAS, ARHGEF2, MYOM1, ITGB6 and CPS1. E2F targets, base excision repair and notch signaling pathway were highly enriched in stiff subtype. Stiff subtype had significantly higher TMB and T cells follicular helper levels than soft subtype, as well as CTLA4, CD276, CD47 and TNFRSF25.

**Conclusions:**

From the perspective of cell membrane tension, we found that stiff and soft tumor subtypes were closely associated with BCR-free survival for PCa patients, which might be important for the future research in the field of PCa.

## Introduction

Many human cancers are closely related to age, especially for urinary tumors [1–4]. Prostate cancer (PCa) is one of the most frequent malignancies in men, which harbors great prevalence in aging males [1, 5, 6]. This phenomenon is exacerbating as the global population ages and the disease burden of this disease is predicted to be increased [1, 5, 7–11]. Standard therapies for clinically localized PCa mainly contain radical prostatectomy (RP) and radical radiotherapy (RT) [5, 12–14]. Unfortunately, a considerable number of patients will develop biochemical recurrence (BCR) despite radical treatments [15]. BCR means the return of measurable PSA, which is associated with high possibility of clinical recurrence, leading to the elevated risks of metastasis and death [6, 13, 14, 16–19]. Currently, the recommended management method for BCR by European Association of Urology is mainly based on prostate-specific antigen doubling time (PSA-DT), Gleason score, International Society of Urological Pathology (ISUP) grade and interval from primary therapy to biochemical failure [15], but there are still defects in the process of clinical application. With the advent of next-generation sequencing, more and more biomarkers for the management of BCR based on transcriptomic data are identified from different perspectives [8, 12, 14, 20–22]. Interestingly, radiomics attracts clinicians’ attention in PCa, such as prostate volume and other traits [23, 24]. However, investigators have not yet considered BCR in PCa from the perspective of membrane tension.

Membrane tension is not a new concept, which refers to the force per unit length acting on a cross-section of membrane, regulating many vital biological processes [25]. Over the past decades, the important role of cell and tissue mechanics in tumorigenesis, progression and metastasis was gradually recognized [26–29]. The decreasing membrane tension of tumor cells is associated with increasing invasion and metastatic capacity, which is proved to be mediated by the Bin/Amphiphysin/Rvs (BAR) family proteins [30]. In contrast, in normal epithelial cells, the underlying mechanism for the homeostatic maintenance of the higher plasma membrane tension is achieved through the membrane-to-cortical attachment regulated by the ezrin, radixin, and moesin (ERM) proteins. If this homeostasis is disrupted, epithelial cells may transform into mesenchymal migratory phenotype powered by BAR proteins, leading to greater capacity for invasion and metastasis [30, 31]. In addition, tumor cells can also feel the changes in exogenous forces through a complex series of cellular sensing and signaling pathway conduction mechanisms, which can affect and regulate the cellular metabolic processes, and then promote the tumor proliferation and progression [28, 32–34]. Therefore, to better reveal the role and mechanism of membrane tension in the progress of tumorigenesis, progression, and metastasis in PCa, we divided PCa patients into 2 subtypes and proposed the concepts of stiff tumor and soft tumor based on 8 identified membrane tension-related genes and the cancer genome atlas (TCGA) database, which provided new perspective and insights for the management of the prognosis and survival of PCa patients.

## Methods

### Data preparation and identification of molecular subtypes

**Fig. 1:**
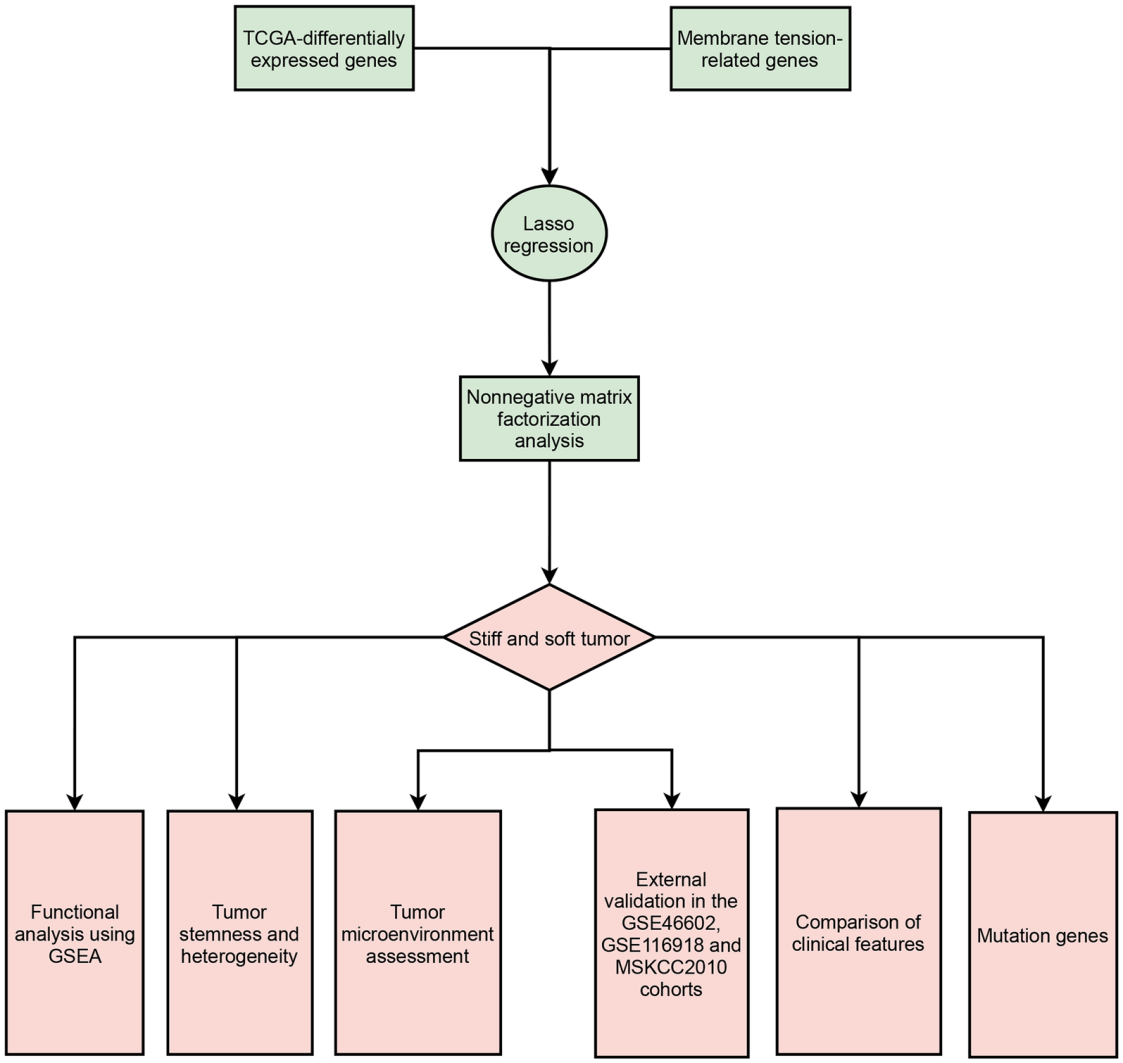
The flowchart of this study

The workflow of our study is illustrated in Fig. 1. We downloaded the gene matrix and clinical data of PCa patients in the TCGA database as the training set from our previous study [20]. For the validation set, we used 2 gene expression omnibus (GEO) datasets (GSE46602 [35], GSE116918 [36]) and MSKCC2010 [37] (http://www.cbioportal.org/). Moreover, we obtained total 44 membrane-related genes from previously published literature [38]. Subsequently, we performed differential analysis between tumor tissue and normal tissue within the TCGA cohort based on R package “limma”. Deferentially expressed genes (DEGs) were defined as llogFCl ≥ 0.4 and p.adj. < 0.05. After the intersection of DEGs and membrane-related genes, we used Lasso regression to identify the final genes. Based these genes, we used nonnegative matrix factorization (NMF) method to divide the patients in TCGA cohorts into two subtypes. We set the cluster number of K index from 2 to 10 and determined the average contour width of the common member matrix using the R package “NMF”. Three external cohorts were used to validate the prognostic value of TCGA subtypes, including GSE46602 [35], GSE116918 [36] and MSKCC2010 [37]. The clinical features between these two subtypes were analyzed.

### Mutation landscape and gene set enrichment analysis (GSEA)

We downloaded RNA-sequencing profiles, genetic mutation and corresponding clinical information of PCa patients from TCGA database (https://portal.gdc.com). The data of mutations were obtained and visualized based on R package “maftools”. We also conducted the differences analysis of mutation frequency between these two subtypes. When performing GSEA analysis by GSEA software (version 3.0) (http://www.gsea-msigdb.org), [39] several gene set databases were used including: “c2.cp.kegg.v7.4.symbols.gmt” and “h.all.v7.4.symbols.gmt” from the molecular signatures database [39, 40]. The minimum gene set was defined as 5 and the maximum gene set was 5000. Resampling was performed as 1000 times. P value < 0.05 and false discovery rate < 0.05 were considered statistically significant.

### Tumor stemness, tumor heterogeneity and tumor immune microenvironment (TIME) analysis

For tumor stemness indexes, we compared differentially methylated probes-based stemness scores (DMPss), DNA methylation-based stemness scores (DNAss), enhancer elements/DNA methylation-based stemness scores (ENHss), epigenetically regulated DNA methylation-based stemness scores (EREG-METHss), epigenetically regulated RNA expression-based stemness scores (EREG.EXPss), RNA expression-based stemness scores (RNAss) [41] and mRNAsi [42] algorithms between two subtypes. For tumor heterogeneity, we compared homologous recombination deficiency (HRD), loss of heterozygosity (LOH), neoantigen (NEO), tumor ploidy, tumor purity, mutant-allele tumor heterogeneity (MATH), tumor mutation burden (TMB) and microsatellite instability (MSI) [43, 44] between two subtypes. All these data could be obtained from our previous study [9]. In addition, for the TIME analysis, we used Cibersortx and ESTIMATE algorithms [45–47] to assess the overall tumor microenvironment and immune components and compared the differences of tumor microenvironment scores and 54 immune checkpoints between these two subtype. Moreover, we calculated the Tumor Immune Dysfunction and Exclusion (TIDE) score to predict potential response of immune checkpoint blockade (ICB) therapy [48] and compared the differences between these two subtypes. All the comparison of differences between these two subtypes were based on the Wilcokson rank sum test.

### Statistical analysis

All analyses were completed through software R 3.6.3 and its suitable packages. For abnormal distribution, we used Wilcoxon test to compare differences between groups. Survival analysis was conducted through log-rank test and presented as Kaplan–Meier curve. Statistical significance was set as two-sided *p* < 0.05. Significant marks were as follows: not significance (ns), *p* ≥ 0.05; *, *p* < 0.05; **, *p* < 0.01; ***, *p* < 0.001.

## Results

### The identification of “stiff tumor” and “soft tumor”

**Fig. 2:**
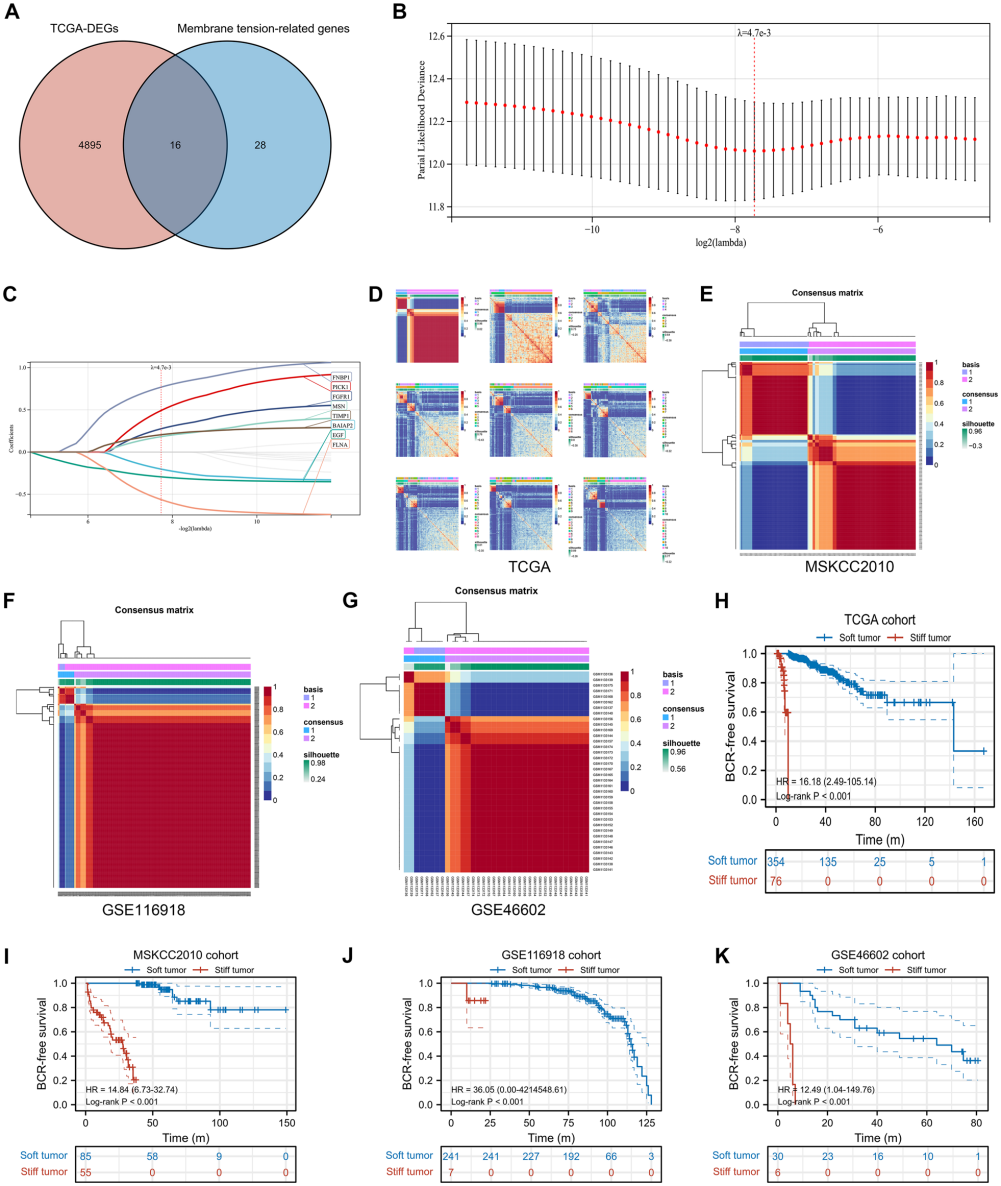
The identification of “stiff tumor” and “soft tumor”. **A**. Venn plot showing the intersection of DEGs and membrane tension-related genes; **B**. identifying 8 genes using Lasso regression analysis; **C**. lasso coefficient profiles of the identifying genes; **D**. the results of heatmaps using NMF algorithm in TCGA cohort; **E**. two subtypes were identified the optimal value using NMF algorithm in MSKCC2010 cohort; **F**. two subtypes were identified the optimal value using NMF algorithm in GSE116918 dataset; **G**. two subtypes were identified the optimal value using NMF algorithm in GSE46602 dataset; **H**. Kaplan–Meier curve showing the survival differences between two subtypes in TCGA cohort; **I**. Kaplan–Meier curve showing the survival differences between two subtypes in MSKCC2010 cohort; **J**. Kaplan–Meier curve showing the survival differences between two subtypes in GSE116918 dataset; **K**. Kaplan–Meier curve showing the survival differences between two subtypes in GSE46602 dataset. DEGs = differentially expressed genes; NMF = nonnegative matrix factorization; TCGA = the cancer genome atlas; BCR = biochemical recurrence; GEO = gene expression omnibus

**Fig. 3:**
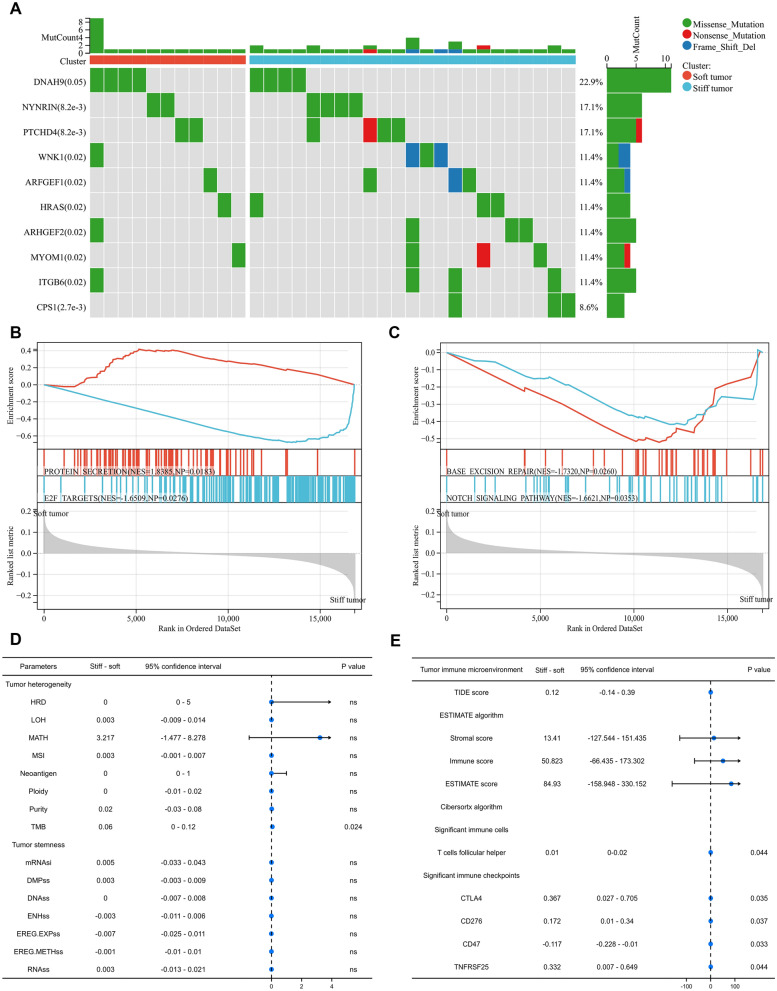
Functional pathways, mutation genes, tumor heterogeneity, tumor stemness and TME evaluation between two subtypes. **A**. Waterfall plot showing the top 10 most commonly mutated genes between two subtypes; **B**, **C**. the functional differences between two subtypes using GSEA; **D**. the differences of tumor heterogeneity and tumor stemness between two subtypes; **E**. the differences of tumor immune microenvironment scores and infiltrating cells between two subtypes. TMB = tumor mutation burden; MATH = mutant allele tumor heterogeneity; MSI = microsatellite instability; HRD = homologous recombination deficiency; LOH = loss of heterozygosity; DMPss = differentially methylated probes-based stemness scores; DNAss = DNA methylation-based stemness scores; ENHss = enhancer elements/DNA methylation-based stemness scores; EREG.METHss = epigenetically regulated DNA methylation-based stemness scores; EREG.EXPss = epigenetically regulated RNA expression-based stemness scores; RNAss = RNA expression-based stemness scores. TIDE = Tumor Immune Dysfunction and Exclusion

The PCa expression profile in the TCGA database included 498 tumor samples and 52 normal samples. The DEGs between tumor samples and normal samples were detected using R package “limma” and finally 4895 genes were obtained to analyze (Fig. 2A). Then, after the intersection of DEGs and membrane-related genes, 16 candidate genes were obtained (Fig. 2A). Through Lasso regression, when lambda (λ) equaled 4.7 ${e}^{-3}$, we obtained the optimal model (Fig. 2B) and 8 identified genes, including FNBP1, PICK1, FGFR1, MSN, TIMP1, BAIAP2, EGF and FLNA (Fig. 2C). Based on these 8 membrane-mediated genes, using NMF algorithm, we divided 430 PCa patients in TCGA cohort into 2 subtypes and named them “stiff tumor” and “soft tumor” based on their biological meanings (Fig. 2D). Similar results were observed in MSKCC2010, GSE116918 and GSE46602 cohorts (Fig. 2E-G). We found the BCR risk of “stiff tumor” was significantly higher than “soft tumor” (HR = 16.18; Fig. 2H). The baseline comparison showed balanced clinical features between “stiff tumor” and “soft tumor” (Table 1). Similar prognostic results were observed in MSKCC2010, GSE116918 and GSE46602 cohorts (Fig. 2I-K). For the differences of mutation landscape between “stiff tumor” and “soft tumor”, the top ten genes were DNAH9, NYNRIN, PTCHD4, WNK1, ARFGEF1, HRAS, ARHGEF2, MYOM1, ITGB6 and CPS1 (Fig. 3A).

**Table 1:** The clinical features between TCGA stiff and soft tumor subtypes for prostate cancer patients

Characteristic	Soft tumor	Stiff tumor	*P* value
Samples	354	76	
Age, median (IQR)	61 (56, 66)	62 (57, 66)	0.526
Gleason score, *n* (%)			0.297
6	32 (7.4%)	7 (1.6%)	
7	175 (40.7%)	31 (7.2%)	
8	50 (11.6%)	9 (2.1%)	
9	97 (22.6%)	29 (6.7%)	
T stage, *n* (%)			1.000
T2	128 (30.2%)	27 (6.4%)	
T3	214 (50.5%)	47 (11.1%)	
T4	7 (1.7%)	1 (0.2%)	
Race, n (%)			0.317
Asian	9 (2.2%)	2 (0.5%)	
Black or African American	45 (10.8%)	5 (1.2%)	
White	288 (69.2%)	67 (16.1%)	
N stage, *n* (%)			1.000
N0	251 (66.9%)	55 (14.7%)	
N1	57 (15.2%)	12 (3.2%)	
Residual tumor, *n* (%)			0.465
No	228 (54.4%)	45 (10.7%)	
Yes	117 (27.9%)	29 (6.9%)	

*IQR* interquartile range

### Functional enrichment analysis, TME evaluation and tumor heterogeneity and stemness

For GSEA, protein secretion was highly enriched in “soft tumor” (Fig. 3B) and E2F targets, base excision repair and notch signaling pathway were highly enriched in “stiff tumor” (Fig. 3B-C). For tumor heterogeneity between “stiff tumor” and “soft tumor”, we found TMB in “stiff tumor” was higher than “soft tumor” with statistical significance (Fig. 3D). In terms of immune checkpoints, the expressions of CTLA4, CD276, CD47 and TNFRSF25 were significantly higher in “stiff tumor” (Fig. 3E). In addition, T cells follicular helper were significantly higher in “stiff tumor” than in “soft tumor” (Fig. 3E).

## Discussion

The metastasis of malignant cells is usually accompanied by alternation of cell mechanical properties. A line of evidence has shown that tumor cells can promote metastasis and change cell metabolism by reducing membrane tension [28, 30, 32–34]. However, PCa appears to deviate from the cell-softening trends described in other cancer models, in which with the increasing of stiffness of PCa cells, the metastasis potential is also increasing [49–53]. In addition, a recent study found that the cell and tissue mechanic characteristics of different metastatic potential cells were diverse, which meant the relationship between the metastatic ability and membrane tension and tissue stiffness of PCa cells was not necessarily linear [54]. These confounding results indicate that microenvironment mechanics are a significant yet nuanced factor in the progression of PCa. Based on these facts and by means of analyzing published sequencing data, we identified 8 membrane-related genes (FNBP1, PICK1, FGFR1, MSN, TIMP1, BAIAP2, EGF and FLNA) and used NMF algorithm to divide PCa patients in TCGA cohort into two subtypes based on these genes.

In PCa, there are many reports on FGFR1, TIMP1, and EGF. For instance, FGFR1 is a kind of fibroblast growth factor (FGF)/FGF receptor, which was overexpressed in PCa [55, 56]. Enhanced FGF signaling plays an important role in tumor progression and drug resistance, which can induce angiogenesis, epithelial-to-mesenchymal transition (EMT), and upregulation of AR [57–59]. In addition, a recent study indicated that TIMP1 was a significant molecular switch that could determine the effects of senescence in PCa [60]. TIMP1 belongs to the TIMP gene family and encodes natural inhibitors of the matrix metalloproteinases (MMPs), which involves in degradation of the extracellular matrix (ECM) [61]. The degradation of ECM plays a wide range of physiological function in angiogenesis, cell repair and remodeling of tissues, while the deficiency of TIMP1 will break the balance between TIMP and MMPs, resulting in the abnormity of angiogenesis, cell proliferation, and apoptosis [61, 62]. In PCa, the loss of TIMP1 can change the senescence-associated secretory phenotype (SASP) of senescent tumor cells through activation of MMPs, thus promoting tumor metastasis [60]. Moreover, EGF is a significant regulatory factor that can induce EMT through increasing expression of transcription factors responsible for reducing E-cadherin and promoting cancer invasion [63]. In summary, the function and role of these three genes in PCa are relatively established. There are a few reports on FNBP1, PICK1, MSN, BAIAP2, and FLNA in PCa. FNBP1(formin-binding protein 1/17) is an actin skeleton-related protein, which is a member of F-Bar/EFC family. Several researches showed that FNBP1 could affect tumor migration and invasion through affecting the formation of filopodia in bladder cancer [64] and breast cancer cells [65]. In addition, a recent study found the loss of FNBP1 could result in the loss of invasive ability in gastric cancer [66]. Remarkably, a pan-cancer analysis based on bioinformatics found that except the metastatic cancer, low expression of FNBP1 was associated with worse prognosis in breast cancer and lung adenocarcinoma, while high expression of FNBP1 was related to the poor prognosis in stomach adenocarcinoma [67]. These studies showed the expression patterns of FNBP1 were associated with the type of tumor and FNBP1 played an important yet complex role of in cancer invasion and metastasis. For PICK1, current understanding of its role in tumor is still limited. PICK1 was reported to involve in regulating the cell to cell junction in epithelial cells, which was associated with tumor invasion and metastasis [68]. In PCa, Dai et al. found the overexpression of PICK1 could suppress the migration and invasion in vitro and bone metastasis in vivo [69]. MSN encodes moesin protein, which belongs to ERM family and appears to function as link protein between plasma membranes and actin-based cytoskeletons [70]. Additionally, Moesin plays a key role in the control of cell morphology, adhesion, and motility [70–72]. The abnormalities of moesin, such as mislocalization, have been proved to be associated with tumor progression in multiple types of tumor [72–74]. Furthermore, moesin plays an important role in the EMT process of breast cancer cells and pancreatic cancer cells, and is currently shown to serve as a potential EMT marker for breast cancer and pancreatic cancer [75–77]. These results presented the huge potential for MSN in cancer research. BAIAP2 and FLNA are rarely reported in cancer. BAIAP2 is also called IRSp53. A bioinformatic study constructed a prognostic model in esophagus squamous cell carcinoma including BAIAP2 [78]. In addition, Funato et al. [79] found IRSp53 could bind to EPs8 to form a complex, thus enhancing the invasive ability of cancer cells. For FLNA, a study reported that FLNA could directly regulate the metastasis and EMT of chemoresistance colorectal cancer cells [80]. Another study found that in bladder cancer, hexavalent chromium could regulate the expression of FLNA to mediate EMT to promote the proliferation and migration and inhibit the apoptosis of bladder cancer cells. In summary, a series of evidence suggests that the 8 identified genes are associated with tumor metastasis and invasion. Based on the evidences and unique biological functions and meanings of these genes, we named the subtype with bad prognosis as “stiff tumor” and the subtype with relatively good prognosis as “soft tumor”.

We found “stiff tumor” had a much worse prognosis than “soft tumor” and speculated it is associated with the differences in enriched pathways between the two subtypes. E2F targets, base excision repair and notch signaling pathway were highly enriched in “stiff tumor” while protein secretion was highly enriched in “soft tumor”. The enrichment of E2F targets in “stiff tumor” means “stiff tumor” cells may have stronger ability of proliferation, because E2F is the key transcription factor for regulating of cell cycle. It is well known that the disorder of cell cycle is one of the important features of cancer cells [81]. Shorter cell cycle implies a faster proliferation rate, correlating with bad prognosis. In addition, a series of evidence suggests the increased expression of base excision repair related genes was associated with rapid recurrence, metastatic dissemination, and decreased patient survival in PCa [82–85], which is consistent with our results. Moreover, a study showed natamycin which was an inhibitor of key BER enzymes DNA polymerase β and DNA Ligase I could significantly inhibit the proliferation of PCa cells in the androgen depleted environment, which furthermore proved that BER signaling pathway played an important role in the bad prognosis in “stiff tumor” [83]. Importantly, we also observed notch signaling pathway enriched in “stiff tumor”. In the physiological state, notch signaling pathway has important role in regulating proliferation, differentiation, cell-fate determination and self-renewal of stem and progenitor cell [86–88]. In the pathological state, the deregulation of notch signaling pathway has been demonstrated in a variety of tumors containing PCa and is associated with tumorigenesis and progression [89–93]. These results strongly support our findings. Interestingly, we found protein secretion was mainly enriched in “soft tumor”. We speculated this may be due to the upregulation of androgen signaling. A study reported compared with hormone-naïve metastatic PCa, hormone-refractory metastatic PCa showed a decrease of androgen signaling and protein biosynthesis [94]. Therefore, we speculated that the higher level of androgen signaling was associated with higher level of protein synthesis and lower malignancy of PCa cells, which was consistent with better prognosis of “soft tumor”.

For tumor heterogeneity, we observed TMB in “stiff tumor” was higher than “soft tumor”. TMB refers to the total number of mutations present in a single tumor specimen, which can be used to reflect immunogenic neoantigens [95]. In many kinds of tumors, higher TMB means tumor cells carry more tumor antigens, which therefore are more vulnerable to be killed by activated immune cells [95, 96]. In PCa, there was a study showing high level of TMB was significantly associated with poor BCR-free survival [97], which was in agreement with our results. In addition, we compared significant immune checkpoints between “stiff tumor” and “soft tumor” and observed the expression of CTLA4, CD276 and TNFRSF25 was higher in “stiff tumor”, while the expression of CD47 was higher in “soft tumor”. CTLA4 is an important inhibitory receptor mainly expressed on T cells, which plays important role in immunosuppression of tumors [98]. Some studies found high expression of CTLA4 was associated with worse prognosis in different kinds of tumors, including PCa [99–102]. CD276 is a newly found molecule in B7 family and probably serves as potential target for multiple kinds of tumors [103]. In PCa, several studies proved that the overexpression of CD276 was associated with bad clinical outcomes in localized PCa [104–107]. In addition, another study found that compared with localized cancer, high expression of CD276 was more frequently observed in metastatic cancer and associated with high disease-specific mortality [108]. These results might explain why “stiff tumor” had worse prognosis to some extent.

There is no doubting that intratumor genetic variability causes sampling bias in gene expression profiles. Furthermore, the microenvironment characteristics may differ in different tumor regions, such as the tumor core and invasive margin. More importantly, whether the identified subtypes in this study could be used in clinical practice in a real-world setting still take a long time.

## Conclusions

In this study, based on 8 membrane tension-mediated genes and their unique biological function, we obtained “stiff tumor” and “soft tumor” subtypes, which provided references and insights to the potential mechanism of invasion and metastasis in PCa and precision medicine.

## Data Availability

The datasets generated and/or analyzed during the current study are available in the TCGA (https://www.cancer.gov/tcga) and GEO (https://www.ncbi.nlm.nih.gov/geo/) repositories.
